# Poor sleep quality correlates with axial symptoms and mood problems in Parkinson’s disease

**DOI:** 10.1080/19585969.2026.2684199

**Published:** 2026-06-15

**Authors:** Seira Taniguchi, Yuta Kajiyama, Emi Shirahata, Yasuyoshi Kimura, Keita Kakuda, Lindun Ge, Kanako Asai, Yoichi Minami, Akifumi Kishi, Nicholas D’Cruz, Hiroki R. Ueda, Moran Gilat, Hideki Mochizuki, Kensuke Ikenaka

**Affiliations:** a Department of Neurology, The University of Osaka Graduate School of Medicine, Suita, Japan; b Department of Rehabilitation Sciences, Neurorehabilitation Research Group (eNRGy), KU Leuven, Leuven, Belgium; c Department of Neurology, Kawasaki Medical School, Kurashiki, Japan; d Department of Neurology, NHO Osaka National Hospital, Osaka, Japan; e Department of Systems Pharmacology, Graduate School of Medicine, The University of Tokyo, Tokyo, Japan; f Osaka Toneyama Medical Center, Toyonaka, Japan

**Keywords:** Parkinson’s disease, sleep, mood disturbances, bed mobility

## Abstract

**Introduction:**

Sleep disturbances and impaired bed mobility are common in Parkinson’s disease (PD). We examined whether objectively measured sleep efficiency is associated with axial motor impairment and explored its potential relationship with cognitive and mood profiles.

**Methods:**

Twenty-eight patients with PD underwent single-night EEG-based sleep monitoring and assessment of nocturnal turning. Partial correlations between sleep metrics and scores on motor, cognitive and mood outcomes were analysed, controlling for age, sex and the MDS-UPDRS 3. Family-wise error (FWE) correction was applied within each sleep predictor.

**Results:**

Poor sleep efficiency correlated with higher MDS-UPDRS axial subscore (*r*_partial_ = −.615) and this association survived stringent correction for multiple comparisons (FWE-*p* = .018). However, its associations with PIGD and clinically rated freezing of gait did not remain significant after the correction. Exploratory analyses showed the relationships of N1 percentage with mood disturbances and REM latency with self-reported freezing of gait, of which only the relationship of N1 percentage with depression score showed a trend towards significance after the correction (*r*_partial_ =.551, FWE-*p* = .074).

**Conclusion:**

Poor objective sleep quality was robustly associated with axial motor severity in PD, independent of overall motor burden. Sleep efficiency may represent a clinically accessible marker of shared brainstem-related pathology underlying axial dysfunction.

## Introduction

Parkinson’s disease (PD) is a complex neurodegenerative disorder characterised by motor and non-motor symptoms. Sleep disturbances are common, reported in up to 98% of people with PD (Roychowdhury and Forsyth 2012). While their aetiology is highly multifactorial (Lajoie et al. 2021; Iranzo et al. 2024), nocturnal motor impairments remain a crucial and debilitating factor in PD (Mirelman et al. 2020). Previously, we reported impaired bed mobility patterns in PD from a biomechanical perspective, finding that axial trunk rotation was compromised (Taniguchi et al. 2022). Consequently, it is not surprising that the majority of patients present with axial symptoms involving turning in bed, postural instability and freezing of gait (FOG). These disturbances markedly diminish quality of life in PD. Growing neuroimaging evidence suggests that these axial symptoms and sleep disturbances may share overlapping neural mechanisms (see reviews Milane et al. 2024; Zitser et al. 2025). As PD progresses, the loss of dopaminergic neurons and reduced striatal dopamine are accompanied by abnormal striatal cholinergic activity (Müller and Bohnen 2013). Beyond the basal forebrain cholinergic nuclei, the pedunculopontine nucleus (PPN; a major component of the mesencephalic locomotor region [MLR]) contains a mixture of cholinergic, glutamatergic and GABAergic neurons (Dautan et al. 2021). The PPN is considered a key node within the ascending reticular activating system (RAS), which regulates arousal and the sleep-wake cycle (Reese et al. 1995). Likewise, the brainstem locus coeruleus, often affected in PD, is a key regulator of sleep-wake states (Breen et al. 2014). With widespread projections to the hippocampus, amygdala, thalamus, cerebellum and neocortex, the locus coeruleus also contributes to motor control and balance (Benarroch 2018). Abnormal regional activation and interactions within these networks contribute not only to postural instability, locomotor deficits and falling in PD (Sherman et al. 2015), but also to non-motor dysfunctions, including rapid eye movement (REM) sleep regulation, cognition and mood (Rye 1997). A review by Lajoie et al. highlights that poor sleep efficiency is one of the most consistently observed objective sleep alterations in PD (Lajoie et al. 2021). However, these associations with motor and non-motor features in PD remain incompletely understood. Several studies have applied polysomnography to objectively capture various primary sleep disorders in PD (Iranzo et al. 2024); however, when focusing on the relationship between sleep and axial motor symptoms such as FOG, most studies have relied on self-reported sleep measures (Milane et al. 2024), which can be vulnerable to recall bias. Critically, the lack of clinical correlations in these studies hampers the clinical management of sleep disturbances in people with PD and limits the development of mechanism-based interventions. Therefore, we aimed to examine the relationships between objective sleep quality and multidimensional motor, cognitive and mood profiles in patients with PD. Based on previous reports (Lajoie et al. 2021; Taniguchi et al. 2022), we specifically focused on whether objectively measured sleep efficiency is associated with axial motor symptoms, while also exploring its potential relationship with cognitive and mood domains. We hypothesised that poor sleep efficiency would be associated with worsening axial symptoms including FOG severity.

## Methods

### Participants

All participants were consecutively enrolled from patients admitted to The University of Osaka Hospital, Japan, from June 2022 to February 2024 and provided written informed consent. Inclusion criteria were: 1) a diagnosis of PD according to the MDS clinical diagnostic criteria, as determined by a movement disorder specialist; and 2) being able to turn in bed and get out of bed, as well as walk ≥ 10 metres without assistance. Exclusion criteria were: 1) diagnosis of a neurological disorder other than PD; and 2) cognitive deficits, as assessed by the Mini-Mental State Examination (MMSE) score < 24. A total of 31 participants met the eligibility criteria and were assessed during a one-week hospitalisation. The study was conducted in accordance with the Declaration of Helsinki and approved by the Research Ethics Committee of the University of Osaka and written informed consent was obtained from all participants prior to study-related activities.

### Multimodal assessments of sleep-related metrics

Sleep quality and nocturnal turning movements were continuously monitored using a combination of wearable devices, and data were recorded throughout the entire period. Other clinical information (levodopa equivalent daily dose [LEDD], the Movement Disorder Society-sponsored revision of the Unified Parkinson’s Disease Rating Scale [MDS-UPDRS] part 1, 2, 3 and 4), and self-reported sleep questionnaires were also collected. Specifically, we used the Epworth Sleepiness Scale (ESS), Pittsburgh Sleep Quality Index (PSQI), both of which are officially recommended for evaluating overall sleep quality and daytime sleepiness in the PD by the MDS Task Force review (Högl et al. 2010) and REM Sleep Behaviour Disorder Questionnaire (RBD-Q), which has been widely used in PD population (Nomura et al. 2011; Liu et al. 2017).

#### Objective sleep parameters

Participants wore an EEG-based sleep monitor (Sleep Profiler PSG2^™^, Advanced Brain Monitoring, USA) equipped with a built-in body position sensor. This device has been recently utilised in studies to evaluate parameters such as sleep efficiency, as well as non-REM and REM sleep metrics (Lucey et al. 2021; Gnarra et al. 2023; Levendowski et al. 2023, 2024; Kataoka et al. 2026) and specific REM sleep abnormalities (Levendowski et al. 2025). Validation studies by Levendowski et al. have demonstrated substantial agreement between this device and gold-standard polysomnography for sleep staging, yielding Kappa values of 0.65–0.82 for Wake, 0.63–0.72 for N2 and 0.72–0.83 for REM sleep in patients with a history of dream enactment behaviour (Levendowski et al. 2023). Additionally, in cohorts including patients with PD and RBD, the automated REM sleep without atonia detection showed strong specificities (88% to 93%) and sensitivities (81% to 86%), alongside high night-to-night consistency (ICC: 0.79–0.84), closely matching expert visual scoring (Levendowski et al. 2025). In the present study, the EEG-based sleep monitor was manually activated by a neurologist when a participant went to bed (lights off) and deactivated upon final awakening (lights on). Importantly, in the calculation of these sleep metrics, any epochs with invalid data (e.g., poor signal quality or artefacts, as identified by the device’s automated algorithm and confirmed by visual inspection by a neurologist) were excluded. To strictly define the actual lying time in bed and exclude wake-time activities during the night (e.g., toilet visits), we cross-referenced the recording with the synchronised sternal accelerometer data (details mentioned in the ‘Kinematics of nocturnal turning in bed’ section) and their sleep diaries. The following variables were estimated: Sleep efficiency (%) was defined as the percentage of time in bed spent asleep, calculated as [(N1 + N2 + N3 + REM)/total time in bed] × 100. A higher percentage of sleep efficiency is a marker of better sleep quality. Total sleep hours, percentage of supine body position, sleep latency, percentage of REM sleep, non-REM stage 1 (i.e., N1), non-REM stage 2 (i.e., N2) and non-REM stage 3 (i.e., N3). A higher percentage of REM sleep indicates more REM relative to total sleep time. REM latency was calculated only for 23 subjects who exhibited REM sleep (REM% > 0). It indicates the amount of time elapsed between the sleep onset and the first REM stage; prolonged REM latency indicates delayed entry into REM sleep. In addition, arousal by movement refers to partial awakening from sleep caused by body movements. Spindle duration refers to waxing-and-waning 10–16 Hz oscillations lasting 0.5–2 s. Percentage of supine body position indicates the percentage of total sleep time a person spends lying on their back. On the following day, participants also completed a sleep diary to record the timing of sleep onset, toilet visits, as well as periods spent sitting or standing. A neurologist reviewed the data to confirm the alignment of sleep epochs between the objective sleep parameters and the participant’s sleep diary. In case of discrepancies, the neurologist (E.S.) consulted another neurologist (Y.K.) to ensure accurate interpretation.

#### Kinematics of nocturnal turning in bed

Sensor-based assessment was conducted using a triaxial accelerometer (Axivity AX3, Axivity Ltd., Newcastle upon Tyne, UK). Our earlier review revealed that the sternum was the most consistent sensor location for detecting nocturnal turning in patients with PD (Taniguchi et al. 2024); thus, a sensor was attached to the sternum with medical tape. To ensure comprehensive capture of the entire night, the accelerometer was pre-programmed to record continuously for 18 h (from 18:00 to 12:00 the following day). Nocturnal rest was defined based on orientation, differentiating between lying and upright positions (e.g. sitting, standing and walking), applying the established threshold of a mean vertical acceleration of < 0.4 g (Mirelman et al. 2020). This physical parameter, cross-referenced with the participants’ sleep diaries, ensured that any upright activities during this extended 18-hour monitoring period were completely excluded from the nocturnal turning analysis. Nocturnal turn parameters, including total turn number and turn velocity, were calculated based on previously established methodology (Mirelman et al. 2020). A turn was defined when the body moved from one static position (e.g., supine, prone, right/left lateral) to a different position with ≥10° change in orientation (Mirelman et al. 2020).

### Motor, cognitive and mood measures

FOG severity was assessed using both self-reported Freezing of Gait Questionnaire (FOG-Q) and clinically rated FOG of MDS-UPDRS item 3.11. In terms of motor assessments, a movement disorder specialist evaluated the MDS-UPDRS part 3 scores and calculated axial subscore (sum of items 3.1, 3.9–3.13), bradykinesia subscore (sum of items 3.2, 3.4–3.8, 3.14), rigidity subscore (sum of items 3.3), postural instability and gait difficulty (PIGD) subscore (the sum of MDS-UPDRS items 2.12, 2.13, 3.10, 3.11 and 3.12). For cognitive assessments, a certified clinical psychologist administered the Montreal Cognitive Assessment (MoCA) for global cognition, the Frontal Assessment Battery (FAB) for executive function, the Benton visual retention test for visual memory and the Stroop interference effect (Stroop test part 3 minus part 1) for task-switching effects. For mood scales, apathy scale (Starkstein et al. 1993; Leentjens et al. 2008), clinical rating of the Hamilton Depression Rating Scale (HAM-D) (Hamilton 1960) and Anxiety subscore (the sum of the HAM-D items 10, 11, 12, 13, 15 and 17) were also collected. To minimise collinearity, we excluded HAM-D insomnia items, item 4 (early in the night), item 5 (middle of the night), item 6 (early hours of the morning) for our correlation analysis.

### Influence of sleep medication

Ten out of 28 participants were administered sleep medication: benzodiazepine (*n* = 6), benzodiazepine combined with another non-benzodiazepine drug (*n* = 1), an orexin receptor antagonist (*n* = 2) and other non-benzodiazepine drugs (*n* = 1). To address the influence of sleep medication intake on clinical associations with sleep metrics, we performed a sensitivity analysis adding sleep medication (yes/no) intake as an additional covariate.

### Statistical analysis

Partial correlations between the sleep-related metrics and motor as well as cognitive measures were analysed, controlling for age, sex and MDS-UPDRS part 3 total scores. Based on our research question, we defined primary outcomes specifically to assess the relationship between sleep efficiency and axial motor impairments: sleep efficiency, axial subscore, REM sleep, REM latency, total turn number and FOG-Q and MDS-UPDRS item 3.11. Secondary outcomes included all other sleep architecture variables: percentages of N1 or N2 or N3 of non-REM, arousal by movement, spindle duration, supine body position and turn velocity and other motor (bradykinesia, rigidity, tremor, PIGD, BBS), cognitive (MoCA, FAB, Stroop, Benton) and mood parameters (apathy, depression, anxiety). Variables that violated the normality assumption were Box–Cox transformed. Multicollinearity was also assessed, and all variance inflation factor (VIF) values were below 1.5. Outliers of continuous variables were defined as values exceeding 1.5 times the interquartile range (IQR) above the third quartile or below the first quartile, and sensitivity analyses were conducted excluding these observations. Statistical analysis was performed using SPSS (version 29) for correlations. A significance level of *p* < .05 was applied. To address the issue of multiple testing, we applied the Bonferroni correction to control the family-wise error (FWE) rate within each sleep predictor.

## Results

### Participant characteristics

Supplementary Table 1 summarises demographics and clinical characteristics for the participants with PD included in the analyses. Of these, 3 participants were excluded due to incomplete data caused by the detachment of the sensor during sleep assessments. The remaining 28 patients were included in the main analysis. Of these, 13 patients had objectively observed FOG while 15 patients did not, based on clinically rated FOG (measured by the MDS-UPDRS item 3.11).

### Objective sleep metrics

Table 1 presents EEG-derived sleep parameters and kinematic measures of nocturnal turning.

**Table 1.** Summary of objective sleep metrics.

	PD participants (*n* = 28)	
Outcomes	Mean ± *SD*	Range (min—max)
EEG parameters		
Sleep efficiency (%)	65.91 ± 16.75	(26.7—88.7)
Wake after sleep onset (WASO; min)	142.71 ± 77.06	(41—339)
Total sleep hours	5.24 ± 1.47	(2.3—8.3)
Sleep latency (min)	21.11 ± 30.43	(1—163)
REM sleep (%; *n* = 27)	10.89 ± 7.53	(0—23)
REM latency (min; *n* = 27)	155.55 ± 94.90	(39—387)
N1 (%)	19.61 ± 15.93	(4.2—69.4)
N2 (%)	53.90 ± 19.38	(11.6—84.1)
N3 (%)	13.43 ± 13.06	(0.0—57.9)
Arousal by movement	4.85 ± 5.84	(0.2—31.4)
Spindle duration (min)	5.45 ± 6.94	(0.0—23.3)
Supine body position (%)	69.68 ± 31.45	(5.1—100.0)
Kinematic parameters of turning		
Total turn number (night)	4.57 ± 2.95	(0—14)
Turn velocity (degree/sec)	7.67 ± 4.06	(2.45—18.21)

Abbreviations: *SD*: standard deviation; REM: rapid eye movement; N1 or N2 or N3: non-rapid eye movement sleep stage 1 or 2 or 3 (%).

### Partial correlations between sleep-related metrics and other outcomes

Table 2 presents the partial correlation coefficients, and corresponding scatterplots are shown in Supplementary Figure 1. Associations involving kinematic turning parameters are reported in the text and Supplementary Figure 1. Data from one PD patient with an outlier in REM% and the corresponding REM latency were excluded from the main analysis.

**Table 2.** Result of partial correlation.

		Motor										Cognition				Mood		
		Turn number	Turn velocity	Brady-kinesia	Rigidity	Tremor	Axial	PIGD	FOG-Q	UPDRS 3.11 (FOG)	BBS	MoCA	FAB	Stroop	Benton	Apathy	Depression	Anxiety
EEG parameters	Sleep efficiency (%)	0.09	0.03	0.37	−0.14	**0.40**	−0.62**	**−0.45**	−0.07	**−0.40**	0.32	−0.32	−0.13	0.08	0.06	0.27	−0.13	−0.01
	WASO	−0.15	0.06	−0.20	0.13	−0.34	0.39	0.35	0.15	0.20	−0.36	0.35	0.04	−0.07	−0.08	−0.21	0.16	0.03
	REM sleep (%)	−0.16	0.38	0.34	−0.18	0.16	−0.27	−0.03	−0.07	−0.03	0.11	0.08	−0.03	−0.03	0.16	0.25	0.04	0.32
	REM latency	−0.11	−0.33	−0.10	0.05	0.01	0.16	0.28	**0.51**	0.22	−0.04	−0.04	−0.01	−0.18	0.06	0.05	0.06	−0.07
	N1 sleep (%)	0.05	0.09	−0.06	−0.13	−0.09	0.22	0.17	0.12	0.06	−0.10	0.08	−0.05	0.14	−0.16	−0.40	0.55*	**0.44**
	N2 (%)	−0.08	−0.13	−0.23	0.11	0.07	0.12	−0.03	−0.08	0.14	0.26	−0.38	0.15	−0.02	0.11	−0.08	−0.29	−0.25
	N3 (%)	0.06	−0.10	0.21	0.03	0.07	−0.29	−0.24	0.08	−0.06	0.09	0.12	−0.07	0.06	0.24	0.25	−0.31	−0.14
	Arousal by movement	0.15	0.24	0.03	−0.20	0.02	0.09	0.20	0.20	−0.01	−0.13	−0.04	−0.20	0.17	0.02	−0.20	0.36	0.12
	Spindle duration	0.05	−0.17	0.01	−0.05	0.13	−0.08	0.01	−0.03	−0.10	0.03	−0.16	0.10	−0.13	0.24	0.15	−0.28	−0.19
	Supine (%)	−0.20	0.19	0.34	−0.22	0.11	−0.28	−0.15	0.26	**−0.50**	0.15	0.05	−0.08	0.29	−0.03	0.12	0.04	0.00

*Notes:* Bolded values indicate statistically significant correlation coefficients (uncorrected *p* < .05), controlling for age, sex, and MDS-UPDRS part 3. Blue indicates inverse correlations, while red indicates positive correlations. Darker colours represent stronger associations, and lighter colours represent weaker associations. Asterisks denote the results of the family-wise error (FWE) correction: ** indicates correlations that fully survived the FWE correction (FWE-*p* < 0.05), and * indicates a trend towards significance after the correction (FWE-*p* < 0.10).

Abbreviations: WASO: wake after sleep onset (min); REM: rapid eye movement; N1 or N2 or N3: non-rapid eye movement sleep stage 1 or 2 or 3 (%); supine (%): supine body position (%); axial: axial subscore (sum of MDS-UPDRS items 3.1, 3.9–3.13); bradykinesia: bradykinesia subscore (sum of MDS-UPDRS items 3.2, 3.4–3.8, 3.14); rigidity: rigidity subscore (sum of MDS-UPDRS item 3.3 subitems); PIGD: postural instability and gait difficulty score (sum of MDS-UPDRS items 2.12, 2.13, 3.10, 3.11 and 3.12); FOG-Q: freezing of gait questionnaire; UPDRS 3.11: MDS-UPDRS item 3.11 (clinically rated freezing of gait); BBS: Berg balance scale; MoCA: Montreal Cognitive Assessment; FAB: Frontal Assessment Battery; Stroop: Stroop interference effect (Stroop test part 3 time − part 1 time); Benton: Benton visual retention test; Apathy: apathy scale; Depression: Hamilton Depression Rating Scale excluding insomnia items; Anxiety: HAM-D anxiety subscore (sum of Hamilton Depression Rating Scale items 10, 11, 12, 13, 15 and 17).

#### Primary outcomes

Poor sleep efficiency was correlated with higher axial subscore (*r*_partial_ = −.615, *p* = .001, FWE-*p* = 0.018; Supplementary Figure 1-A) and with higher PIGD subscores (*r*_partial_ = −.450, *p* = .024, FWE-*p* = 0.406; Supplementary Figure 1-B), as well as with worse clinically rated FOG (*r*_partial_ = −.399, *p* = .048, FWE-*p* = 0.818; Supplementary Figure 1-C). Moreover, fewer turns were associated with worse self-reported FOG (*r*_partial_ = −.423, *p* = .040, FWE-*p* = 0.633; Supplementary Figure 1-D). In addition, longer REM latency was associated with worse self-reported FOG (*r*_partial_ =.509, *p* = .031, FWE-*p* = 0.530; Supplementary Figure 1-E).

#### Secondary outcomes

Higher sleep efficiency was associated with higher tremor scores (*r*_partial_ =.402, *p* = .046, FWE-*p* = 0.358; Supplementary Figure 1-F). A greater percentage of N1 sleep was associated with higher depression score (*r*_partial_ =.551, *p* = .004, FWE-*p* = 0.074; Supplementary Figure 1-G), as well as with a higher anxiety subscore (*r*_partial_ =.439, *p* = .028, FWE-*p* = 0.478; Supplementary Figure 1-H); this association remained significant after excluding outliers (*r*_partial_ =.564, *p* = .005, FWE-*p* = 0.238). A lower percentage of supine position during sleep was associated with worse clinically rated FOG (*r*_partial_ = −.502, *p* = .010, FWE-*p* = 0.136; Supplementary Figure 1-I).

Importantly, these significant correlation patterns for the primary and secondary outcomes remained consistent even after excluding the MDS-UPDRS part 3 (i.e., controlling only for age and sex). In summary, under the most conservative multiple comparison correction (FWE), the association between poor sleep efficiency and a higher axial subscore fully survived the FWE correction (FWE-*p* = 0.018), while the associations between a greater percentage of N1 sleep and higher depression scores (FWE-*p* = 0.074) showed a trend towards significance after the correction.

#### Sensitivity analysis

In sensitivity analysis, all significant associations remained significant after additional adjustment for sleep-medication intake: sleep efficiency with axial subscore (*r*_partial_ = −.698, *p* = .0001); PIGD subscores (*r*_partial_ = −.500, *p* = .013) and with FOG item 3.11 (*r*_partial_ = −.423, *p* = .040); REM latency with self-reported FOG (*r*_partial_ =.537, *p* = .022); turn number with FOG-Q (*r*_partial_ = −.434, *p* = .038); percentage of N1 sleep with depression score (*r*_partial_ =.557, *p* = .005) and with anxiety subscore (*r*_partial_ =.430, *p* = .036); percentage of supine position during sleep with FOG item 3.11 (*r*_partial_ = −.524, *p* = .009).

## Discussion

We explored associations between objective sleep metrics and their clinical correlates, encompassing multidimensional profiles of motor, cognition and mood. The primary finding of this study was the robust association between poor sleep efficiency and axial motor severity. Additional exploratory associations were observed between REM latency and FOG severity and between N1 sleep and depressive symptoms.

### Poor sleep efficiency was robustly associated with worse axial motor features

We found that poor objective sleep efficiency was related to worse motor features, with no associations with cognition or mood. These motor features of axial, PIGD and FOG, that were associated with poor sleep efficiency are commonly classified as the axial symptoms. From a kinematic perspective, reduced axial mobility contributes to difficulty turning in bed, particularly overnight when dopaminergic medication effects are lowest. Hypothetically, if axial trunk movements contributed substantially to poor sleep efficiency, we would expect to observe associations between polysomnographic sleep metrics and kinematic indices of nocturnal turning in our PD cohort. However, we did not observe such associations.

This absence suggests that poor sleep efficiency in PD is not merely a direct biomechanical consequence of nocturnal turning difficulties, but rather reflects the complex and multifactorial nature of sleep disturbances in PD. These include the intrinsic degeneration of neural structures that modulate sleep, the detrimental effects of dopaminergic medications and coexisting sleep disorders such as circadian rhythm dysfunction, insomnia, restless legs syndrome and obstructive sleep apnoea (Lajoie et al. 2021; Iranzo et al. 2024). Therefore, future studies should consider these factors for a more comprehensive understanding. Unexpectedly, we further observed that poor objective sleep efficiency was associated with better tremor scores, even though severe limb tremors are typically expected to cause poor sleep quality. One possible explanation is that our cohort presented with only mild-to-moderate tremor severity (range 0–9 out of 32). Given the absence of severe tremors in our sample, future studies should recruit cohorts with a more diverse range of tremor severity to clarify this relationship.

### Associations of FOG severity with fewer nocturnal turns and prolonged REM latency

Fewer nocturnal turns (Mirelman et al. 2020) and prolonged REM sleep latency (Dodet et al. 2024) have been previously reported in the PD population. In the present study, we observed that these deficits were potentially associated with worse self-reported FOG, although these findings should be interpreted cautiously as they did not survive FWE correction. As noted earlier, both FOG and reduced nocturnal turns are axial symptoms reflecting axial motor control, in which cholinergic mesencephalic neurons play a key role. Abnormal hyperactivation of the PPN/MLR has been reported in PD (Garcia-Rill et al. 2015), and fMRI studies have shown both abnormally increased PPN/MLR activation (Snijders et al. 2011) and stronger functional connectivity with other regions in PD patients with FOG (Gilat et al. 2015). In addition, basal forebrain cholinergic neurons during non-REM sleep are thought to facilitate the transition to REM sleep (Vazquez and Baghdoyan 2001), and degeneration of this basal forebrain cholinergic system occurs early in PD (Bohnen and Albin 2011). Collectively, widespread cholinergic pathology affecting both brainstem and basal forebrain networks likely underpins the observed impaired bed mobility, FOG and difficulty initiating REM sleep (manifesting as prolonged REM latency).

### Relationship between mood symptoms and higher percentage of N1 sleep

In our exploratory analyses, worse mood symptoms (depression and anxiety) were initially associated with a higher percentage of N1 sleep. However, after applying FWE correction, only the association with depression score showed a trend towards significance. Depression is thought to originate in the limbic brain regions (Zhao et al. 2025). In PD, a positron emission tomography (PET) study demonstrated that the locus coeruleus degeneration is associated with the severity of depression, resulting from a reduction in noradrenergic innervation of the limbic system (Remy et al. 2005). Taken together, the locus coeruleus pathology and its interactions with limbic regions might partly underlie this preliminary observation of the relationship between worse depressive symptoms and N1 sleep in PD patients.

### Lack of associations with cognition and apathy

Despite the well-reported role of the basal forebrain cholinergic system in cognition (Müller and Bohnen 2013), we found no association between sleep metrics and cognitive scores in the current study. This could be explained by a large meta-analysis in neurotypical adults showing that mood is more affected by sleep deprivation than either cognitive or motor aspects (Pilcher and Huffcutt 1996). In PD, subcortical limbic structures—especially the amygdala and hippocampus—are affected early, preceding widespread prefrontal cognitive impairment and supporting extensions of the Braak staging model (Braak et al. 2006). Notably, unlike depressive symptoms, apathy did not associate with any sleep metric. This may reflect its status as a distinct neuropsychiatric syndrome in PD: whereas depression can appear in the prodromal phase, apathy often emerges in early-stage disease alongside mild cognitive impairment (Poewe et al. 2017) and may be more linked to fronto-striatal dysfunction than to sleep physiology. Although our current findings highlight a robust association with axial motor severity and a potential association with depressive symptoms, these results do not preclude an influence of cognitive function on sleep. Future studies focusing exclusively on the relationship with cognition are needed.

## Limitations

This study has several limitations. First, it was a single-arm design with a modest sample size and no group comparisons, which may limit the generalisability of our findings. In addition, although patients were consecutively enrolled, only those who provided informed consent were included, and thus selection bias cannot be excluded. Future multi-centre studies with larger cohorts are needed to validate these associations. Second, this was a cross-sectional study; longitudinal analyses in larger cohorts are needed to investigate potential causal relationships. Third, we adjusted for common confounders (age, sex and motor severity) and also performed a sensitivity analysis by adjusting for sleep-medication intake; however, residual confounding factors such as specific PD subtypes, psychiatric conditions and concurrent PD-related sleep disorders (e.g., sleep apnoea, periodic limb movement disorder) may still be present. Future studies should incorporate these factors to better isolate independent associations. Fourth, although the Sleep Profiler was previously validated in PD (Levendowski et al. 2025), its standalone use without video recording prevented verification of possible dream enactment behaviour, possibly leading to inaccurate outcomes of REM sleep. Therefore, these results should be interpreted with caution. Furthermore, as clinical apathy scales in PD often overlap with symptoms of depression, fatigue, or cognitive impairment, these specific results require careful interpretation. Additionally, while we utilised MDS-recommended sleep questionnaires (e.g., PSQI, ESS), we did not employ PD-specific sleep scales such as the Parkinson’s Disease Sleep Scale 2nd version or the Scale for Outcomes in Parkinson’s Disease-Sleep. Similarly, we used the RBD-Q, which is a screening tool and cannot definitively confirm the presence of true RBD without video-polysomnography. Finally, the neurobiological basis of sleep disturbance in PD is complex. Although we highlighted key aspects of motor, cognitive and mood domains, future work integrating brain imaging (e.g., structural/functional MRI or PET) alongside sleep and a more varied set of kinematic measures is needed for a clearer understanding.

## Conclusion

Objective sleep metrics captured by EEG and accelerometry revealed the clinical associations with worse axial motor and depressive mood problems in PD. These findings support the idea of shared brainstem mechanisms, implicating the locus coeruleus and basal forebrain cholinergic pathology, which are affected in PD. Future longitudinal studies with larger cohorts are needed to examine causal relationships and evaluate whether improving sleep can ameliorate these motor and mood symptoms.

## Supplementary Material

Supplementary_material_.docx

## Data Availability

The data that support the findings of this study are available from the corresponding author upon reasonable request.
