# Supplementary material for: Poor sleep quality correlates with axial symptoms and mood problems in Parkinson’s disease
Source: Dialogues Clin Neurosci. 2026 Jun 15;28(1):250–8. doi: 10.1080/19585969.2026.2684199 (PMC13270875; doi:10.1080/19585969.2026.2684199)
Supplement: Supplementary_material_.docx [file TDCN_A_2684199_SM0528.docx]

**Supplementary Table 1. Demographics and clinical assessment**

| Variables | PD participants (n = 28) | |
| --- | --- | --- |
| Age (years) | 65.61 | ± 8.89 |
| Sex | n = 12 female /16 male | |
| Disease duration (years) | 8.79 | ± 5.81 |
| Hoehn and Yahr (stage 1/2/3/4/5) | n = 2/10/13/3/0 | |
| Use of sleep medication (yes/no) | n = 10/18 | |
| LEDD (mg/day) | 815.89 | ± 468.21 |
| MDS-UPDRS part1 total | 9.44 | ± 5.19 |
| MDS-UPDRS part2 total | 13.15 | ± 6.62 |
| MDS-UPDRS part3 total | 25.14 | ± 11.10 |
| MDS-UPDRS part4 total | 6.52 | ± 5.05 |
| ESS | 10.48 | ± 5.78 |
| PSQI | 8.44 | ± 3.98 |
| RBD-Q | 6.11 | ± 3.13 |
| *Motor* |  |  |
| Bradykinesia subscore | 12.54 | ± 6.78 |
| Rigidity subscore | 4.61 | ± 3.07 |
| Tremor subscore | 2.14 | ± 2.40 |
| Axial subscore | 5.86 | ± 4.42 |
| PIGD subscore | 6.29 | ± 4.04 |
| Freezing of gait questionnaire (FOG-Q) | 13.07 | ± 4.45 |
| MDS-UPDRS item 3.11 (0/1/2/3/4 points) | n = 15/8/4/0/1 | |
| Berg Balance Scale (BBS) | 50.32 | ± 14.22 |
| *Cognition* |  |  |
| MMSE | 28.30 | ± 1.76 |
| MoCA | 23.82 | ± 3.83 |
| FAB | 15.44 | ± 2.42 |
| Benton visual retention test | 14.36 | ± 6.40 |
| Stroop interference effect (s) | 37.89 | ± 62.07 |
| *Mood* |  |  |
| Apathy ^#1^ | 13.78 | ± 6.44 |
| Depression ^#2^ | 3.32 | ± 3.51 |
| Anxiety ^#3^ | 1.89 | ± 2.21 |

Values denote mean ± standard deviation

Abbreviations: LEDD = levodopa equivalent daily dose, MDS-UPDRS = Movement Disorder Society-sponsored revision of the Unified Parkinson's Disease Rating Scale, axial subscore (sum of sub-items 3.1, 3.9–3.13), bradykinesia subscore (sum of sub-items 3.2, 3.4–3.8, 3.14), rigidity subscore (sum of sub-items 3.3), PIGD = postural instability and gait difficulty score (the sum of MDS-UPDRS items 2.12, 2.13, 3.10, 3.11, and 3.12), ESS = Epworth Sleepiness Scale, PSQI = Pittsburgh Sleep Quality Index, RBD-Q = REM Sleep Behavior Disorder Questionnaire, MMSE = Mini-Mental State Examination Total Score, MoCA = Montreal Cognitive Assessment, FAB = Frontal Assessment Battery, Stroop interference effect (s) = Stroop test part 3 time − part 1 time, ^#1^ Apathy = Apathy Evaluation Scale, ^#2^ Depression = Hamilton Depression Rating Scale excluding insomnia items, and ^#3^ Anxiety = Anxiety subscore (the sum of the Hamilton Depression Rating Scale items 10, 11, 12, 13, 15, and 17).


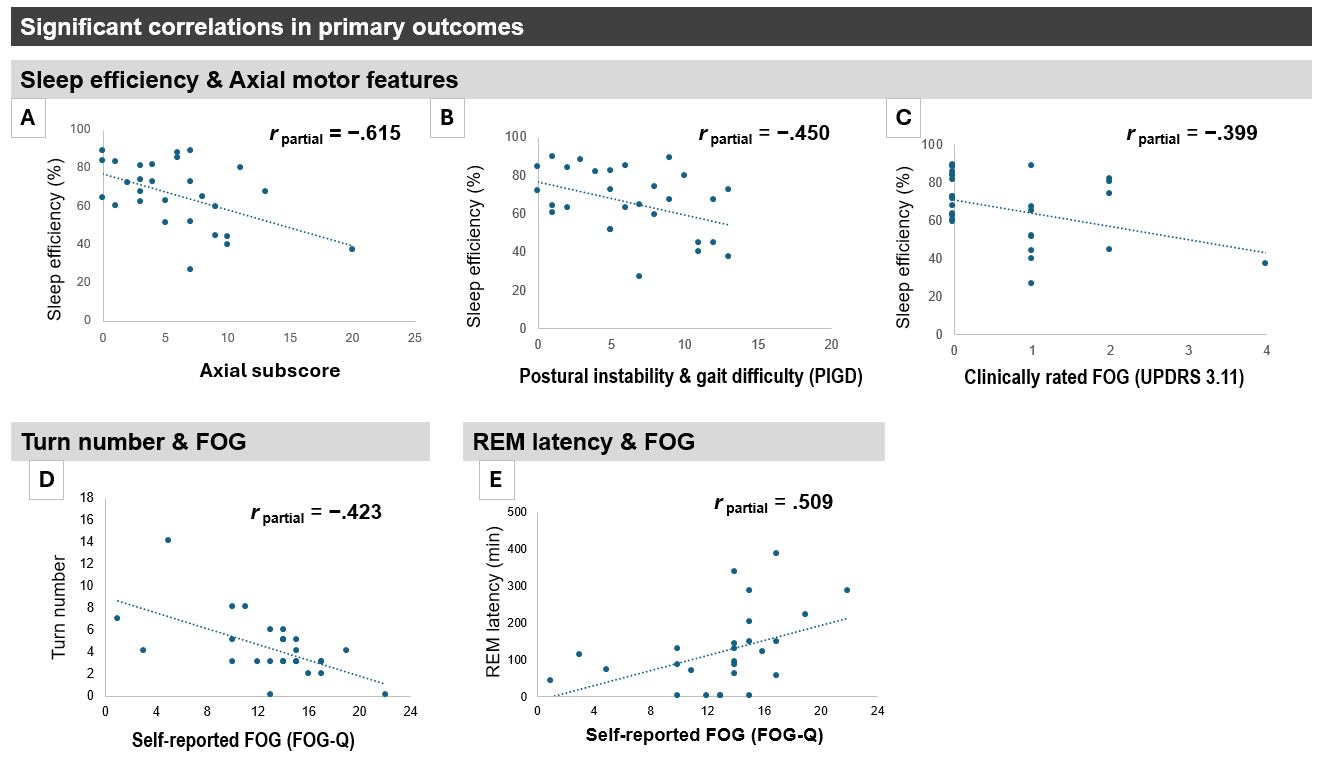


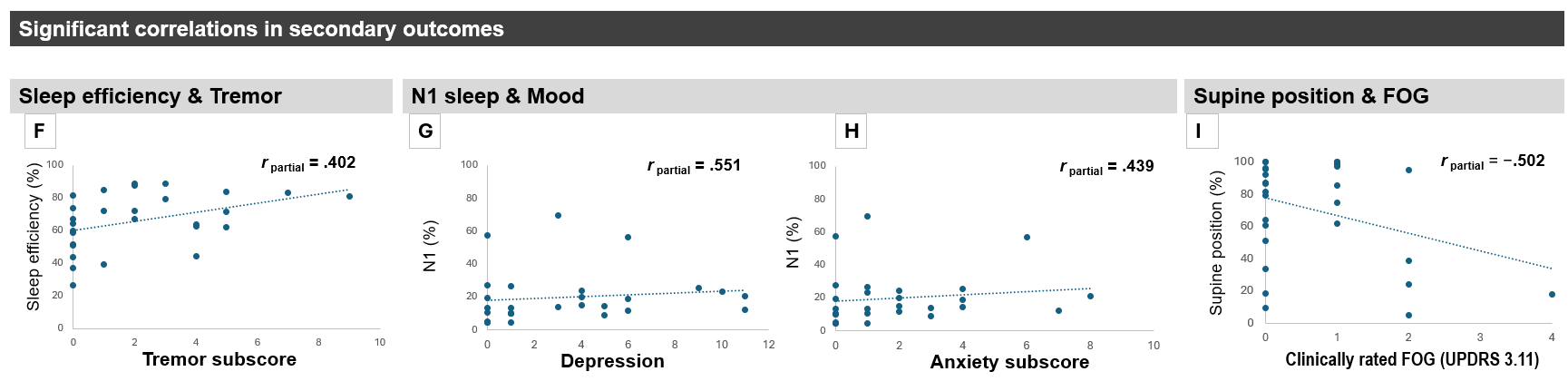


**Supplementary Figure 1. Scatter plots of the significant correlations (uncorrected)**

The scatter plots display the results of the partial correlations between sleep metrics and various measures using raw, untransformed data (i.e., before any Box–Cox transformation and without covariate adjustment). r _partial_ represents the partial correlation coefficients controlling for age, sex, and MDS-UPDRS part 3. For more details, refer to the result section.

Abbreviations: REM = Rapid eye movement, N1 = non rapid-eye movement sleep stage 1, UPDRS = Movement Disorder Society-sponsored revision of the Unified Parkinson's Disease Rating Scale, axial subscore (sum of sub-items 3.1, 3.9–3.13), PIGD = postural instability and gait difficulty score (the sum of MDS-UPDRS items 2.12, 2.13, 3.10, 3.11, and 3.12), Depression = Hamilton Depression Rating Scale excluding insomnia items, Anxiety = HAM-D anxiety subscore (the sum of the Hamilton Depression Rating Scale items 10, 11, 12, 13, 15, and 17), FOG-Q = Freezing of gait questionnaire, UPDRS 3.11 = MDS-UPDRS item 3.11 (clinically rated freezing of gait).
